# Antimicrobial effect of benzbromarone combined with colistin against multidrug-resistant bacteria

**DOI:** 10.3389/fmed.2025.1665514

**Published:** 2025-09-10

**Authors:** Feng Wang, Hongjun Li, Xiaoqiang Bao

**Affiliations:** Department of Respiratory and Critical Care Medicine, Shin-an International Hospital, Jiaxing, Zhejiang, China

**Keywords:** COPD, bacterial resistance, colistin, benzbromarone, combination therapy

## Abstract

**Introduction:**

Drug-resistant respiratory pathogens in COPD patients pose a major therapeutic challenge. Colistin is a last-resort treatment for drug-resistant infections. But emerging resistance and hepatorenal toxicity limit its use, which highlights the need for combination strategies to improve its efficacy and safety. This study investigated the antibacterial effect of the combination of benzbromarone and colistin against drug-resistant pathogens associated with COPD.

**Methods and Results:**

In this study, MIC determination experiments revealed that respiratory pathogens from COPD patients exhibited high resistance rates to several conventional antibiotics. Furthermore, the checkerboard assay showed that benzbromarone alone had no antibacterial effect but markedly lowered the MIC of colistin, with all FICI values <0.5. Further growth curve and time kill curve analysis showed that colistin combination with benzbromarone significantly enhanced bacterial growth inhibition and exerted bactericidal activity. Additionally, the antibacterial mechanism showed that colistin combined with benzbromarone synergistically enhanced bacterial membrane damage, promoted ROS accumulation, and inhibited ATP production, thereby exerting stronger bactericidal effects. Moreover, validation using the Galleria mellonella larval infection model demonstrated that the benzbromarone-colistin combination provided effective protection against infections caused by multidrug-resistant Gram-negative bacteria, with larval survival rates reaching up to 70%, which was significantly higher than that observed with monotherapy.

**Discussion:**

This study revealed that benzbromarone enhanced the antibacterial activity of colistin against COPD-associated drug-resistant pathogens, exhibiting a significant synergistic effect while effectively reducing the required colistin dosage. These findings provide a promising alternative approach for antimicrobial therapy in drug-resistant COPD infections and contribute to the exploration of new antibacterial applications for non-antibiotic drugs.

## Introduction

Chronic obstructive pulmonary disease (COPD) is a chronic inflammatory pulmonary disease characterized by persistent airflow limitation and typically caused by prolonged smoking or exposure to harmful gases or particulate matter (1). According to the Global Burden of Disease study, COPD has become one of the leading causes of mortality worldwide, with its prevalence increasing annually (2). The pathophysiological characteristics of COPD include chronic airway inflammation and airway remodeling, involving the regulation of various cytokines and inflammatory mediators (3). The persistent inflammatory state weakens the airway defense mechanisms, leading to immune system dysfunction and rendering COPD patients more susceptible to infections by various pathogens, particularly bacterial respiratory infections (4). Chronic obstructive pulmonary disease Bacterial infections, especially those affecting the lower respiratory tract, are among the primary triggers of acute exacerbations in COPD patients (5). These infections not only worsen the disease condition but also contribute to increased hospitalization rates and mortality. Consequently, the impact of bacterial infections on COPD has become a critical focus of research. Among COPD patients, Gram-negative bacteria such as *Pseudomonas aeruginosa* (*P. aeruginosa*), *Klebsiella pneumoniae* (*K. pneumoniae*), and *Acinetobacter baumannii* (*A. baumannii*) are commonly identified pathogens (6, 7). These Gram-negative bacteria employ multiple pathogenic mechanisms, including extracellular polysaccharides, endotoxins, and secreted proteases, to invade pulmonary tissue and exacerbate local inflammatory responses (8). In particular, *P. aeruginosa* poses a significant clinical challenge due to its robust biofilm formation capacity and high antibiotic resistance, often leading to refractory pulmonary infections (9). This bacterium not only directly induces pulmonary infections but also exacerbates airway inflammation in COPD through immune evasion mechanisms, further accelerating the deterioration of lung function (10). Studies have demonstrated that the presence of Gram-negative bacteria is closely associated with acute exacerbations of COPD, and their increasing antimicrobial resistance presents significant challenges for clinical management (11, 12). Therefore, the development of more effective therapeutic strategies targeting respiratory drug-resistant infections is of paramount importance.

The pharmacological treatment of COPD primarily relies on bronchodilators, inhaled corticosteroids, and other adjunctive medications (13). However, in cases of bacterial infections, antibiotic therapy becomes crucial. Commonly used antibiotics include broad-spectrum agents such as β-lactams, aminoglycosides, fluoroquinolones, and macrolides, which are generally effective against common COPD-associated pathogens (14, 15). However, with the widespread and prolonged use of antibiotics, the emergence of antibiotic-resistant bacteria has become a significant challenge in clinical treatment. Bacterial resistance, particularly to commonly used antibiotics, has evolved into a major global public health concern (16). COPD patients, who often receive prolonged antibiotic therapy during hospitalization, are at an increased risk of exposure to multidrug-resistant bacteria, particularly Gram-negative pathogens such as *P. aeruginosa* and *K. pneumoniae* (17, 18). Resistant pathogens such as *P. aeruginosa* not only exhibit intrinsic resistance to conventional antibiotics but also enhance their resistance through the production of β-lactamases, aminoglycoside-modifying enzymes, and other resistance mechanisms (19–22). The emergence of antibiotic-resistant bacteria significantly reduces the efficacy of antimicrobial treatment in COPD patients, exacerbates infections, and accelerates disease progression, posing substantial challenges to clinical management. Currently, combination therapy strategies have gained considerable attention, as the concurrent use of conventional antibiotics with adjuvant compounds that possess antimicrobial activity may enhance antibacterial efficacy and mitigate the development of resistance (23, 24). Therefore, focusing on the efficacy, specificity, safety, and clinical feasibility of combination therapeutic strategies holds great promise for providing more effective treatment options for bacterial infections.

Benzbromarone is a drug primarily used for the treatment of gout. As a potent uricosuric agent, it lowers serum uric acid levels by inhibiting urate reabsorption (25, 26). In recent years, benzbromarone has garnered significant research interest due to its potential in the field of antimicrobial therapy (27, 28). Studies have demonstrated that benzbromarone exhibited notable inhibitory activity against certain Gram-positive bacteria, such as *Staphylococcus aureus* (*S. aureus*) and Streptococcus species, by altering bacterial cell membrane stability (29). In our study, we further investigated the synergistic effects of benzbromarone in combination with colistin against COPD-associated respiratory pathogens, particularly multidrug-resistant (MDR) Gram-negative bacteria. COPD patients frequently suffer from infections caused by MDR Gram-negative pathogens, including *P. aeruginosa*, *K. pneumoniae*, and *A. baumannii*, which significantly compromise the effectiveness of conventional antibiotic treatments. Colistin, a last-resort antibiotic with potent activity against Gram-negative bacteria, has regained clinical attention in recent years due to its efficacy against drug-resistant pathogens. However, monotherapy with colistin poses challenges such as the emergence of bacterial resistance and potential toxic side effects. Therefore, our research explored the combination of benzbromarone and colistin to assess their synergistic effects against COPD-related respiratory pathogens. This work provided new ideas and strategies for the development of therapies targeting respiratory infections caused by multidrug-resistant Gram-negative bacteria.

## Results

### Antibiotic resistance of COPD-associated respiratory pathogens

Before evaluating the antibacterial effects of benzbromarone in combination with colistin against COPD-associated respiratory pathogens, it is essential to first determine the susceptibility of these pathogens to benzbromarone alone and other commonly used antibiotics. To achieve this, we randomly selected and tested laboratory-isolated and preserved COPD-associated bacterial strains for their minimum inhibitory concentrations (MICs), assessing their resistance profiles against benzbromarone and clinically relevant antibiotics. The results demonstrated that benzbromarone exhibited varying MIC values against Gram-positive bacteria, including *S. aureus*, *Staphylococcus epidermidis*, *Streptococcus agalactiae*, and *Streptococcus pneumoniae*, with MICs ranging from 2 to 8 μg/mL (Table 1). In contrast, Gram-negative pathogens such as *P. aeruginosa*, *K. pneumoniae*, and *A. baumannii* exhibited MIC values exceeding 128 μg/mL, indicating a high level of resistance to benzbromarone (Table 1). Most Gram-positive strains displayed sensitivity to levofloxacin (MIC range: 0.5–16 μg/mL), whereas they exhibited higher MIC values for penicillin (4–32 μg/mL) (Table 1). Some isolates also demonstrated significant resistance to tetracycline and azithromycin, with MICs reaching up to 64 μg/mL (Table 1). Meanwhile, the tested Gram-negative strains also exhibited elevated MIC values for colistin, with *K. pneumoniae* and *A. baumannii* showing MICs in the range of 2–16 μg/mL, while *P. aeruginosa* exhibited an MIC as high as 32 μg/mL, indicating substantial resistance (Table 1). Furthermore, all tested Gram-negative strains displayed high MICs for levofloxacin, tetracycline, azithromycin, and penicillin, with some isolates exhibiting MICs as high as 256 μg/mL for azithromycin and 128 μg/mL for tetracycline, highlighting their multidrug-resistant (MDR) nature (Table 1). Overall, these findings suggested that Gram-negative bacteria exhibited a more severe resistance profile, whereas Gram-positive bacteria retained partial susceptibility to certain antibiotics. These results underscore the significant challenges associated with treating COPD-related infections and emphasize the urgent need for novel drugs and combination therapy strategies to enhance treatment efficacy.

**Table 1:** MICs of drugs for strains used in the study.

Species	Source	MIC (μg/mL)						
		Ben	LEV	TET	AZM	PCN	CLI	COL
*S. aureus* ATCC29213		4	0.5	1	1	2	0.25	64
*E.coli* ATCC25922		>128	0.0625	2	64	128	64	0.5
*S. pneumoniae* 11412	China (Zhe Jiang)	4	2	8	16	32	8	128
*S. pneumoniae* 11013	China (Zhe Jiang)	2	0.5	32	32	32	16	64
*S. pneumoniae* 12476	China (Zhe Jiang)	8	1	16	64	16	8	128
*S. aureus* 2241	China (Zhe Jiang)	8	0.5	16	32	4	16	64
*S. aureus* 2254	China (Zhe Jiang)	4	16	32	8	8	32	128
*S. aureus* 2278	China (Zhe Jiang)	4	8	64	16	16	16	128
*S. epidermidis* 1152	China (Zhe Jiang)	8	4	32	16	16	8	64
*S. epidermidis* 1168	China (Zhe Jiang)	4	0.5	16	16	8	16	64
*S. agalactiae* 41	China (Zhe Jiang)	2	0.5	16	32	4	32	128
*S. agalactiae* 44	China (Zhe Jiang)	2	1	8	32	8	16	128
*P. aeruginosa* 10170	China (Zhe Jiang)	>128	8	32	64	128	128	16
*P. aeruginosa* 10147	China (Zhe Jiang)	>128	32	64	256	128	64	32
*P. aeruginosa* 15081	China (Zhe Jiang)	>128	32	16	32	128	64	8
*K. pneumoniae* 12136	China (Zhe Jiang)	>128	16	128	256	256	128	16
*K. pneumoniae* 11411	China (Zhe Jiang)	>128	8	16	64	128	64	8
*K. pneumoniae* 15412	China (Zhe Jiang)	>128	16	8	16	128	64	4
*A. baumannii* 50114	China (Zhe Jiang)	>128	32	32	256	128	256	8
*A. baumannii* 52135	China (Zhe Jiang)	>128	8	16	128	256	128	4
*A. baumannii* 51127	China (Zhe Jiang)	>128	16	16	128	256	128	2

Ben, Benzbromarone; LEV, Levofloxacin; TET, Tetracycline; AZM, Azithromycin; PCN, Penicillin; CLI, Clindamycin; COL, colistin.

### Benzbromarone in synergy with colistin to fight resistant Gram-negative bacteria

**Figure 1:**
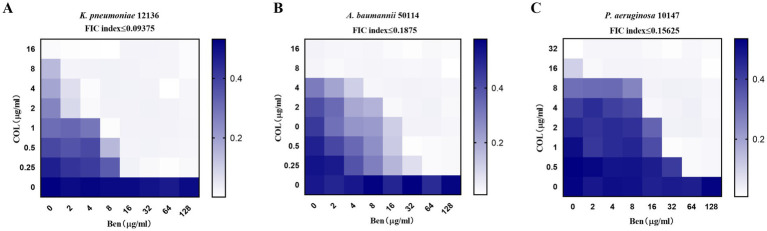
Benzbromarone enhanced the activity of colistin. **(A–C)** Microdilution checkerboard analysis showing the combined effect of benzbromarone and colistin against multidrug-resistant Gram-negative bacteria (*K. pneumoniae* 12136, *A. baumannii* 50114, and *P. aeruginosa* 10147, respectively). All FIC index was less than 0.5.

Benzbromarone was reported to be a membrane-active compound that disrupted Gram-positive bacterial membranes but showed no direct activity against Gram-negative bacteria. Colistin, a last-line agent targeting the Gram-negative outer membrane, was increasingly compromised by resistant strains. We therefore hypothesized that benzbromarone could serve as an adjuvant to potentiate colistin activity, not for its clinical indication but for its unique membrane-active properties. To further evaluate the synergistic antibacterial activity of benzbromarone and colistin against drug-resistant Gram-negative bacteria, we selected three highly colistin-resistant strains and employed the checkerboard dilution method to assess the combination inhibitory effects. The results indicated that for *K. pneumoniae*12136, heatmap analysis showed that even at relatively high concentrations of colistin (≥4 μg/mL) or Benzbromarone (≥32 μg/mL), bacterial growth remained robust, suggesting a high level of resistance to both drugs when used individually (Figure 1A). However, when colistin and benzbromarone were combined, bacterial growth was significantly inhibited, as indicated by a progressive lightening of color in the heatmap, demonstrating a strong synergistic antibacterial effect (Figure 1A). This finding suggested that benzbromarone enhanced colistin’s antibacterial activity against *K. pneumoniae* 12136. For *A. baumannii* 50114, which exhibited slightly lower resistance to colistin than *K. pneumoniae* 12136, bacterial growth was already significantly inhibited at colistin = 8 μg/mL. However, only when benzbromarone reached ≥32 μg/mL did colistin sensitivity markedly improve (Figure 1B). In contrast, *P. aeruginosa* 10147 exhibited the highest level of resistance to both colistin and benzbromarone. Nevertheless, a certain degree of synergistic effect was still observed, with bacterial growth being noticeably restricted at colistin = 0.5 μg/mL and benzbromarone = 64 μg/mL (Figure 1C). In short, these findings suggested that benzbromarone significantly enhanced the antibacterial activity of colistin against resistant Gram-negative bacteria, highlighting its potential role as a colistin potentiator in the treatment of drug-resistant bacterial infections.

### Benzbromarone and colistin combination significantly inhibits the growth of multidrug-resistant Gram-negative bacteria

**Figure 2:**
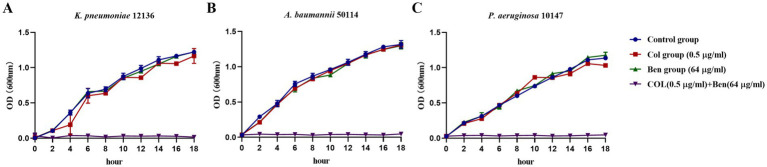
Benzbromarone combined with colistin inhibited the growth of multidrug-resistant Gram-negative bacteria. **(A–C)** Growth curves of benzbromarone combined with colistin against multidrug-resistant Gram-negative bacteria (*K. pneumoniae* 12136, *A. baumannii* 50114, and *P. aeruginosa* 10147, respectively).

To further confirm whether benzbromarone can restore the inhibitory effect of colistin on highly colistin-resistant Gram-negative bacteria and validate its potential as a colistin potentiator, we monitored bacterial growth dynamics by measuring OD600 values to assess the enhancement effect of benzbromarone on colistin activity. The combined concentrations applied in the growth inhibition and related experiments were selected according to the synergistic concentration ranges determined by the checkerboard assay. Growth curve analysis of *K. pneumoniae* 12136 (Figure 2A), *A. baumannii* 50114 (Figure 2B), and *P. aeruginosa* 10147 (Figure 2C) showed that in the control group (blue), colistin group (red), and Benzbromarone group (green), bacterial growth trends were similar, with OD600 values increasing over time. This indicated that monotherapy with either 0.5 μg/mL colistin or 64 μg/mL benzbromarone had limited inhibitory effects on bacterial growth. However, in the combination treatment group (purple), bacterial growth was completely suppressed, with OD600 values remaining close to zero, demonstrating that benzbromarone significantly enhanced colistin’s antibacterial activity against all three strains. Although *P. aeruginosa* 10147 exhibited the highest resistance to colistin, benzbromarone still enhanced its antibacterial effect, and their synergistic action was sufficient to inhibit bacterial growth. Overall, this experiment further confirmed that benzbromarone significantly enhanced colistin’s antibacterial activity against highly resistant Gram-negative bacteria and exerted strong synergistic effects on *K. pneumoniae*, *A. baumannii*, and *P. aeruginosa*. These results aligned with the findings from the checkerboard assay, supporting the potential application of benzbromarone as a colistin potentiator.

### Synergistic bactericidal effect of benzbromarone and colistin against multidrug-resistant Gram-negative bacteria

**Figure 3:**
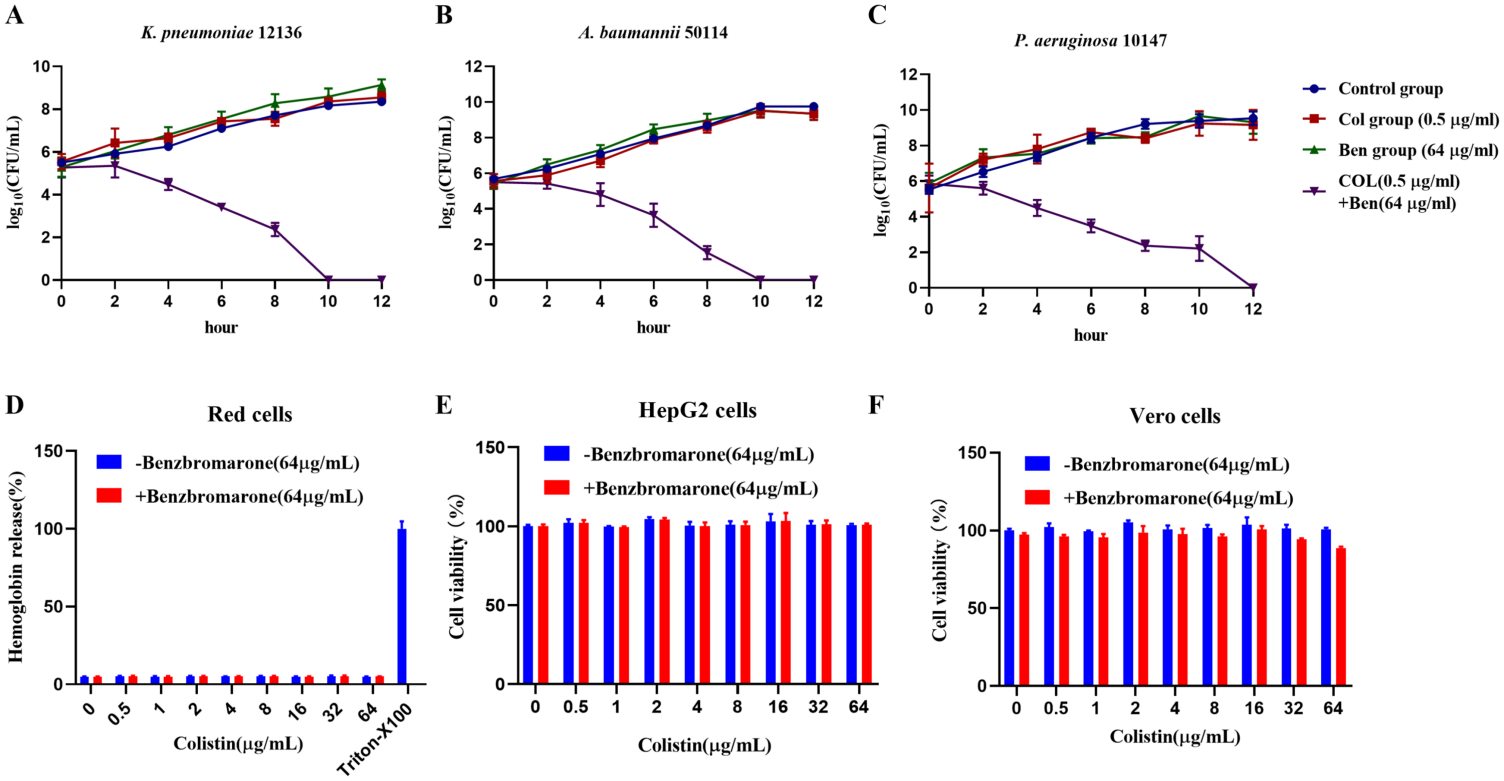
Benzbromarone combined with colistin to kill multidrug-resistant Gram-negative bacteria. **(A–C)** Kill curves of control treatment, benzbromarone, colistin, and combination against multidrug-resistant Gram-negative bacteria (*K. pneumoniae* 12136, *A. baumannii* 50114, and *P. aeruginosa* 10147, respectively) were shown. **(D)** Results of the red blood cell hemolysis assay; **(E)** Cell viability of HepG2 cells; **(F)** Cell viability of Vero cells. All data were means and standard deviations of three independent experiments.

Understanding the mode of action of antimicrobial agents—whether bactericidal (killing bacteria) or bacteriostatic (inhibiting bacterial growth without killing)—is crucial for clinical applications. While our previous OD600 growth curve experiments demonstrated that benzbromarone enhanced colistin’s antibacterial activity against resistant Gram-negative bacteria. OD600 primarily reflected total biomass and does not distinguish between bactericidal and bacteriostatic effects. Therefore, we employed the colony-forming unit (CFU/mL) counting method to dynamically monitor the viable bacterial population over 12 h, aiming to elucidate the mechanism of action when Benzbromarone and Colistin are used in combination. The results showed that for *K. pneumoniae* 12136, the CFU count in the control, colistin-only, and benzbromarone-only groups continued to increase over time, maintaining log10 CFU/mL levels between 9 and 10 at 12 h (Figure 3A). This indicated that monotherapy with 0.5 μg/mL colistin or 64 μg/mL benzbromarone had no significant bactericidal effect. However, in the combination treatment group, the bacterial count began to decline after 2 h and dropped to the detection limit by 10 h, demonstrating a strong bactericidal effect (Figure 3A). Similarly, for *A. baumannii* 50114, the colistin-only and benzbromarone-only groups showed bacterial growth patterns similar to the control (blue). In contrast, the combination treatment group (purple) exhibited a log10 CFU/mL count below 1 at 10 h, indicating a potent bactericidal effect (Figure 3B). For *P. aeruginosa* 10147, bacterial counts in the combination treatment group approached the detection limit at 12 h, suggesting a significant bactericidal effect (Figure 3C). However, the killing rate was slower compared to *K. pneumoniae* and *A. baumannii*, indicating potential differences in bacterial susceptibility to the combination therapy. In addition, the evaluation of hemolytic activity on red blood cells and cytotoxicity on mammalian cells showed that colistin, either alone or in combination with benzbromarone (64 μg/mL), did not cause significant hemolysis within the concentration range of 0–64 μg/mL (Figure 3D). The hemolysis rate was comparable to that of the negative control, and only the Triton X-100 group exhibited marked hemolysis (Figure 3D). These results indicate that the combination of benzbromarone and colistin does not increase hemolytic toxicity. In HepG2 cells (Figure 3E) and Vero cells (Figure 3F), colistin alone or combined with benzbromarone showed no significant effect on cell viability at different concentrations, with cell viability remaining at a high level. These findings not only confirmed the potential of Benzbromarone as a colistin potentiator but also provided a novel therapeutic strategy for treating infections caused by colistin-resistant bacteria.

### Benzbromarone enhances colistin-induced bacterial membrane permeability

**Figure 4:**
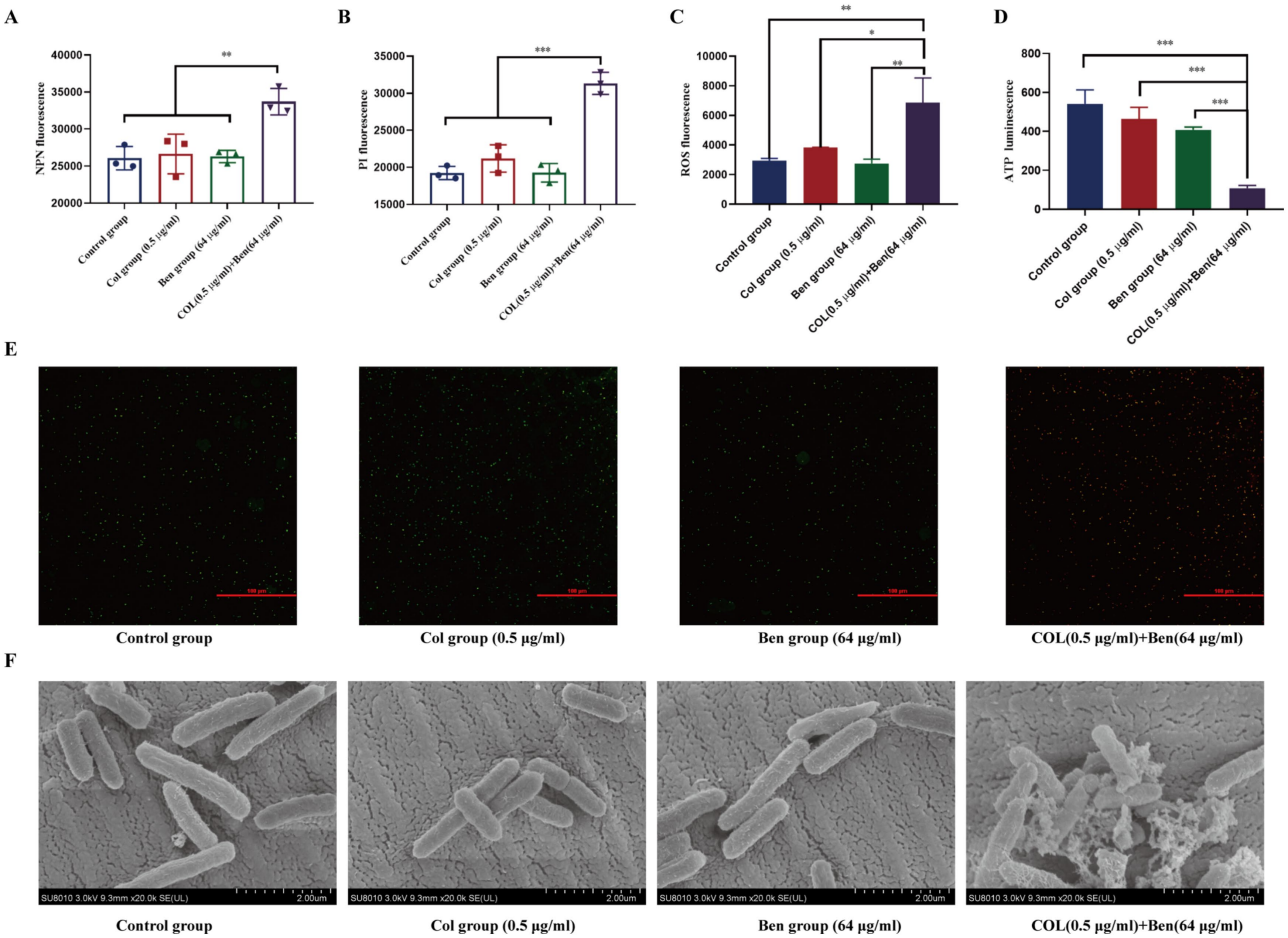
Benzbromarone combined with colistin destroyed the stability of bacterial membrane. Determination of membrane permeability of *E. coli* ATCC25922 using NPN **(A)** and PI **(B)**. **(C)** Intracellular ROS levels measured by fluorescence intensity. **(D)** ATP levels after drug treatment. **(E)** SYTO 9/PI double staining combined with confocal microscopy was used to observe the viability of bacteria. Green fluorescence indicates live bacteria, and red indicates dead bacteria. **(F)** Representative SEM images showing morphological changes of bacterial cells in each treatment group (scale bar: 2 μm). All data are means and standard deviations from at least three independent experiments. Independent sample comparisons were analyzed using one-way ANOVA. **p < 0.01, ***p < 0.001.

Colistin, a polymyxin antibiotic, exerts its bactericidal effect primarily by binding to the lipopolysaccharide (LPS) of Gram-negative bacteria, disrupting membrane integrity and leading to bacterial death. However, resistant bacteria often modify LPS structure, enhance outer membrane barrier function, or activate efflux systems to reduce colistin’s bactericidal efficacy. Our previous findings demonstrated that benzbromarone enhanced colistin’s bactericidal activity against resistant Gram-negative bacteria, but the underlying mechanism remains unclear. To investigate this, we used 1-N-phenylnaphthylamine (NPN) fluorescence assay and propidium iodide (PI) fluorescence assay to assess changes in bacterial outer membrane and cytoplasmic membrane permeability, respectively. NPN fluorescence intensity analysis revealed no significant differences among the control, colistin-only, and benzbromarone-only groups, indicating that neither 0.5 μg/mL colistin nor 64 μg/mL benzbromarone alone significantly disrupted the bacterial outer membrane (Figure 4A). However, the combination treatment group exhibited a significantly higher NPN fluorescence signal (*p* < 0.01), suggesting that benzbromarone enhanced colistin-induced outer membrane damage, facilitating NPN penetration and thereby increasing membrane permeability (Figure 4A). Similarly, PI fluorescence analysis showed no significant differences among the control, colistin-only, and benzbromarone-only groups, indicating that neither colistin nor benzbromarone alone significantly compromised the bacterial cytoplasmic membrane. In contrast, the combination treatment group displayed the highest PI fluorescence signal, with a statistically significant difference compared to other groups (*p* < 0.001) (Figure 4B). This result indicated that benzbromarone enhanced colistin-induced cytoplasmic membrane damage, allowing PI to penetrate and bind to intracellular DNA, thereby increasing fluorescence intensity. Meanwhile, ROS fluorescence assays (C) revealed that combined treatment significantly promoted the accumulation of intracellular reactive oxygen species, reaching levels far higher than those observed in the single-drug groups, suggesting that the combined drug effect can trigger a strong oxidative stress response (Figure 4C). Furthermore, ATP content assays demonstrated that combined treatment with colistin and benzbromarone resulted in a significant decrease in intracellular ATP levels and a severe inhibition of energy metabolism (Figure 4D). Moreover, SYTO 9/PI double-stained bacterial samples were further imaged using a laser confocal microscope (Figure 4E). The results showed that green fluorescence (live bacteria) was predominant in the drug-alone treatment group, while red fluorescence (dead bacteria) increased significantly in the combined drug treatment group, suggesting that combined drug treatment has a strong destructive effect on bacteria, achieved through membrane disruption (Figure 4E). Scanning electron microscopy images further revealed changes in the bacterial surface ultrastructure: the cell surface of the control group was smooth and intact, while the cell surface showed slight wrinkling and damage after treatment with Colistin or Benzbromarone alone (Figure 4F). In the combined treatment group, the cell morphology was severely deformed, with a rough surface and accompanied by ruptures, indicating that the cell structure was severely damaged (Figure 4F). Taken together, these results indicate that the combination of Colistin and Benzbromarone can synergistically enhance bacterial membrane damage, promote ROS accumulation, and inhibit ATP production, resulting in a stronger killing effect on bacteria.

### Benzbromarone enhanced the antibacterial activity of colistin sensitivity against multidrug-resistant Gram-negative bacteria *in vivo*

**Figure 5:**
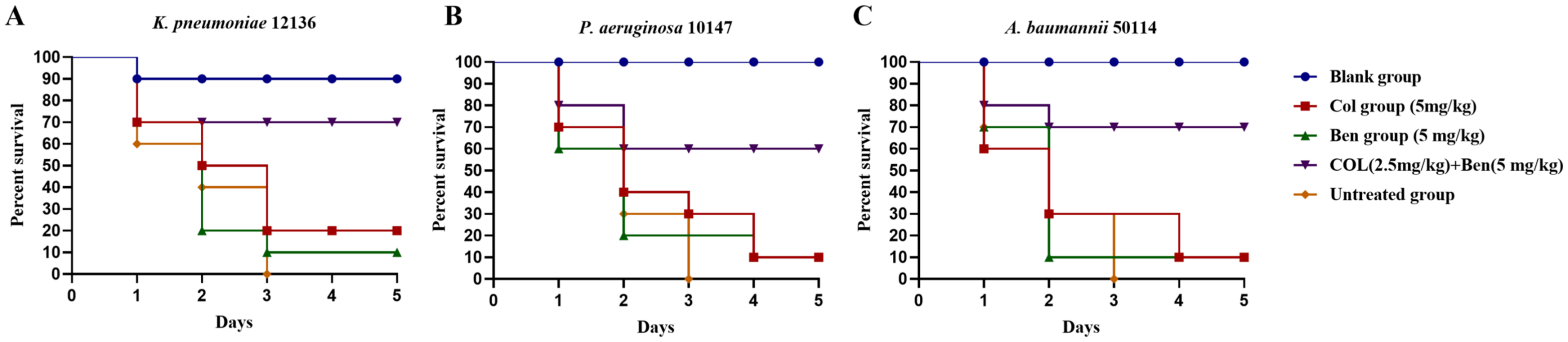
Effects of benzbromarone and colistin combination therapy in Galleria mellonella larvae infection model. **(A–C)** Survival rate of blank control, untreated control, benzbromarone, colistin, and combination against multidrug-resistant Gram-negative bacteria (*K. pneumoniae* 12136, *A. baumannii* 50114, and *P. aeruginosa* 10147, respectively) were shown. There were 10 larvae in each group.

Colistin serves as the last-line therapy against multidrug-resistant (MDR) Gram-negative bacterial infections. However, the emergence of colistin-resistant strains presents a significant clinical challenge. Our previous *in vitro* studies demonstrated that benzbromarone (Ben) significantly enhanced the antibacterial activity of colistin (Col) against MDR Gram-negative bacteria. However, in vitro evidence alone is insufficient to evaluate its protective effects in living organisms. Therefore, we employed the *Galleria mellonella* (wax moth larvae) infection model to assess the *in vivo* antibacterial efficacy of colistin and benzbromarone combination therapy, using survival rate (%) as the primary outcome measure. Experimental results showed that in *K. pneumoniae* (Figure 5A), *P. aeruginosa* (Figure 5B), and *A. baumannii* (Figure 5C) infection models, untreated larvae (Untreated group) succumbed entirely within 3 days, indicating the high lethality of these MDR Gram-negative pathogens. Monotherapy with colistin (5 mg/kg) or benzbromarone (5 mg/kg) slightly improved survival rates, but the five-day survival rate remained below 30%, suggesting that single-drug treatment provides limited protection against severe infections. Interestingly, although benzbromarone alone did not display antibacterial activity *in vitro*, treatment of infected larvae with benzbromarone (5 mg/kg) improved survival compared with the untreated group. This protective effect may be related to its reported anti-inflammatory and antioxidative activities rather than direct bacterial killing. In contrast, the combination therapy group (colistin 2.5 mg/kg + benzbromarone 5 mg/kg) exhibited significantly improved survival rates, reaching approximately 70% for *K. pneumoniae*, 60% for *P. aeruginosa*, and 70% for *A. baumannii* infections. These findings indicated that benzbromarone enhanced the *in vivo* antibacterial activity of colistin, allowing a 50% dose reduction (from 5 mg/kg to 2.5 mg/kg) while maintaining high survival rates. This dose reduction may also mitigate colistin-associated adverse effects. Overall, these results provided further support for benzbromarone as a potential colistin adjuvant, offering experimental evidence for its application in combination therapy against MDR Gram-negative bacterial infections.

## Discussion

Combination therapy has emerged as an important strategy to address antimicrobial resistance. Compared with monotherapy, drug combinations can broaden the antibacterial spectrum, reduce the required dose of individual agents, enhance bactericidal activity, and delay the emergence of resistance (30). In particular, combining membrane-active adjuvants with last-line antibiotics offers a rational approach to overcome intrinsic or acquired resistance mechanisms (31). In this study, we selected the combination of benzbromarone and colistin based on the complementary nature of their mechanisms of action. Previous studies have shown that benzbromarone, as a membrane-active compound, can disrupt the cell membrane of Gram-positive bacteria but exhibits no direct antibacterial activity against Gram-negative bacteria (29). In contrast, colistin is one of the last-line antibiotics for the treatment of multidrug-resistant Gram-negative infections, targeting the bacterial outer membrane. However, the frequent emergence of colistin-resistant strains has greatly limited its efficacy as monotherapy. Based on this background, we hypothesized that benzbromarone could act as an adjuvant to enhance the antibacterial activity of colistin and help overcome resistance. It should be noted that benzbromarone was not selected because of its clinical indication, but rather for its unique membrane-active properties, which make it a rational candidate for exploring colistin-potentiating strategies. This study therefore evaluated the antibiotic resistance profiles of respiratory pathogens associated with COPD and further examined the antibacterial effects of benzbromarone–colistin combination therapy. Antimicrobial susceptibility testing revealed that respiratory pathogens in COPD patients displayed high resistance rates to multiple conventional antibiotics, with *P. aeruginosa*, *K. pneumoniae*, and *A. baumannii* showing particularly severe resistance to penicillins and aminoglycosides. Colistin remained one of the few antibiotics with good activity against these resistant strains. This finding was consistent with global reports of rising antimicrobial resistance in COPD patients in recent years (17, 32), highlighting the need for clinicians to consider the widespread transmission of resistant bacteria and its impact on antibiotic therapy. Furthermore, the resistance data from this study support the necessity of combination therapy, emphasizing the importance of identifying suitable potentiators to be used in conjunction with colistin to reduce its required dosage, enhance therapeutic efficacy, and minimize the survival and spread of resistant bacteria.

Benzbromarone is a classic uricosuric agent primarily used for the treatment of hyperuricemia (26). However, recent studies have suggested that certain non-antibiotic drugs may possess potential antimicrobial activity or enhance the efficacy of antibiotics (33, 34). This study systematically explored, for the first time, the antibacterial effects of the combined use of benzbromarone and colistin against COPD-related pathogens. While benzbromarone itself exhibited no significant antibacterial activity, its combination with colistin significantly reduced the latter’s MIC. Moreover, the combination of benzbromarone and colistin demonstrated synergistic effects (FICI < 0.5). This phenomenon may be related to benzbromarone enhancing the interaction between colistin and bacterial lipopolysaccharides (LPS), thereby increasing bacterial membrane permeability and facilitating the bactericidal action of colistin. Further membrane permeability assays confirmed that benzbromarone could restore colistin’s ability to disrupt the outer membrane of Gram-negative bacteria. Previous studies have shown that benzbromarone enhanced antimicrobial activity by affecting membrane stability in Gram-positive bacteria (29), and our study further suggested that it similarly influence bacterial membrane structures in Gram-negative bacteria through an indirect mechanism. Notably, benzbromarone’s direct effect in Gram-positive bacteria primarily involved disrupting membrane stability, whereas in Gram-negative bacteria, its effect relied on colistin’s outer membrane-disrupting activity, indicating mechanistic differences across bacterial types. Additionally, since the outer membrane of Gram-negative bacteria often serves as a barrier to antimicrobial agents (35), benzbromarone’s ability to enhance colistin’s permeability is particularly important, potentially making it an ideal potentiator for colistin and other antimicrobial peptides. Time-kill assays further confirmed this phenomenon, showing a significant reduction in bacterial colony counts within a shorter time in the combination group, with a faster bactericidal rate than colistin alone. In addition to membrane disruption, our results demonstrated that the combination of colistin and benzbromarone markedly induced intracellular ROS accumulation and reduced ATP levels in Gram-negative bacteria. Excessive ROS can damage cellular components, including DNA, proteins, and lipids, while ATP depletion impairs essential metabolic processes and energy-dependent repair systems. Together, these effects further compromise bacterial viability and may explain the enhanced bactericidal activity observed with the combination treatment. These findings suggest that benzbromarone not only potentiates colistin by destabilizing the bacterial membrane but also contributes to oxidative stress and energy metabolism collapse, thereby providing a multifaceted mechanism underlying the observed synergy. This finding not only provided a novel strategy for treating drug-resistant bacterial infections, but also offered experimental evidence for optimizing colistin dosage. However, this study did not fully elucidate the precise mechanism of benzbromarone’s action. Future studies should integrate molecular biology techniques to identify its potential targets and signaling pathways.

Currently, infections caused by multidrug-resistant (MDR) Gram-negative bacteria have become a major challenge in clinical treatment, particularly due to the resistance of MDR *P. aeruginosa*, *K. pneumoniae*, and *A. baumannii* to multiple antibiotics, severely limiting therapeutic options (36). Colistin remains a crucial treatment choice for such resistant infections due to its unique mechanism of action. However, its dose-dependent toxicity restricts clinical use (37, 38). Previous studies have shown that certain non-antibiotic drugs, such as auranofin (39) and flavomycinn (40), can enhance colistin’s antibacterial activity. This study further demonstrated that benzbromarone could serve as a potential colistin potentiator, allowing for a reduction in the effective dosage of colistin, thereby mitigating adverse effects associated with high-dose administration. Compared to other potentiators, benzbromarone exhibited a distinct advantage, as its synergistic effect has been validated across multiple resistant strains without observed antagonism, highlighting its strong potential for combination therapy. Additionally, benzbromarone were a clinically approved uricosuric agent widely used in the treatment of gout and hyperuricemia (26), yet studies on its antimicrobial activity remain limited. This study is the first to reveal benzbromarone’s ability to enhance colistin’s antibacterial effects and experimentally confirm its role in lowering the MIC of colistin-resistant bacterial strains. These findings not only expand the pharmacological profile of benzbromarone but also provide a new direction for the development of antibiotic potentiators. Furthermore, COPD patients often receive long-term antibiotic treatments, leading to a high prevalence of drug-resistant respiratory pathogens (41, 42). The findings of this study provide a new perspective for future therapeutic strategies against COPD-related multidrug-resistant bacterial infections, potentially improving clinical outcomes in resistant bacterial infections. However, despite demonstrating the synergistic antibacterial effect of benzbromarone and colistin, this study has certain limitations. First, the findings are primarily based on *in vitro* experiments and an *in vivo* model using Galleria mellonella larvae. The efficacy of this combination therapy has yet to be validated in mouse models or clinical studies. Future research should include in vivo studies to assess its effectiveness in complex physiological environments. Second, while this study confirmed that benzbromarone reduces colistin’s MIC, it did not directly investigate whether benzbromarone alleviates colistin’s toxicity. Further research is needed to evaluate its safety and clinical applicability in vivo. Moreover, considering the variability in pathogen species and resistance profiles among COPD patients, the clinical applicability of this combination therapy requires further investigation.

In conclusion, this study is the first to report that benzbromarone enhances colistin’s antibacterial activity against COPD-associated multidrug-resistant Gram-negative bacteria, demonstrating a synergistic antimicrobial effect. This finding not only provides a new strategy for combination therapy against resistant bacterial infections but also offers experimental evidence for optimizing colistin use. However, further in vivo studies and clinical trials are necessary to comprehensively evaluate the potential application of benzbromarone in antimicrobial resistance treatment and to elucidate its underlying molecular mechanisms, paving the way for future antimicrobial drug development.

## Materials and methods

### Bacterial strains and reagents

This study utilized COPD-associated respiratory pathogens, including *S. aureus*, *Streptococcus pneumoniae*, *Staphylococcus epidermidis*, *Streptococcus agalactiae*, *K. pneumoniae*, *P. aeruginosa*, and *A. baumannii*. All bacterial strains were isolated from COPD patients and identified by 16S rRNA sequencing before being stored in the laboratory. *S. aureus* ATCC29213, *E.coli* ATCC25922, HepG2 (ATCC HB-8065) and Vero (ATCC CCL-81) cells were obtained from the American Type Culture Collection (ATCC, Manassas, VA, USA). Bacteria were cultured in Luria-Bertani (LB) agar, Tryptic Soy Broth (TSB), or Mueller-Hinton (MH) broth at 37°C with shaking at 200 rpm. Benzbromarone (CAS: 3562-84-3, 99.81%) was purchased from MCE, dissolved in DMSO (Sigma, D2650) to prepare a stock solution (10 mg/mL), and stored at −20°C. Colistin (CAS: 1264-72-8, ≥96.0%) was obtained from MCE (HY-B0076A) and dissolved in sterile distilled water (10 mg/mL). The remaining antibiotics were purchased from Shanghai Yuanye Biological Co., Ltd. with a purity greater than 95%. The control group contained 1% DMSO. N-Phenyl-1-naphthylamine (NPN, CAS: 90-30-2) was purchased from Sigma-Aldrich (104067) and prepared as a 4 mg/mL ethanol solution. Propidium iodide (PI, CAS: 25535-16-4) was obtained from Beyotime Biotechnology and prepared as a 1 mg/mL sterile water solution, stored at 4°C protected from light. Defibrinated sheep erythrocytes were purchased from Yuanye Biotechnology Co., Ltd.

### Minimum inhibitory concentration (MIC) determination

MIC values were determined using the microbroth dilution method based on CLSI (Clinical and Laboratory Standards Institute) guidelines (43). Briefly, bacterial cultures were grown in liquid media at 37°C to the logarithmic phase (OD600 ≈ 0.5). The cultures were then diluted to 5 × 10^5^ CFU/mL in sterile MH broth. A 96-well plate was prepared by adding 100 μL of the bacterial suspension to each well, followed by 100 μL of serially two-fold diluted drug solutions. The plate was incubated at 37°C for 18 h. Pure MHB medium was used as a negative control, while untreated bacterial samples served as positive controls. Each compound was tested in triplicate, and experiments were repeated at least three times. The MIC was defined as the lowest drug concentration at which no visible bacterial growth was observed.

### Checkerboard dilution assay for drug combination

The fractional inhibitory concentration index (FICI) was determined using the checkerboard dilution method (44). Antibacterial agents were serially diluted along the horizontal axis, while drugs was diluted along the vertical axis. Overnight bacterial cultures were transferred and grown to an OD600 of approximately 0.5, washed with PBS, and adjusted to 1 × 10^6^ CFU/mL in MHB medium. A 96-well plate was prepared by adding 100 μL of MHB medium containing drugs to each well. After incubation at 37°C for 18 h, the MIC was recorded as the lowest drug concentration at which no visible bacterial growth was observed. The FICI was calculated using the formula: FIC index = MIC_AB_/MIC_A_ + MIC_BA_/MIC_B_. Where MIC_A_ was the MIC of compound A alone, MIC_AB_ was the MIC of compound A in combination with compound B, MIC_B_ was the MIC of compound B alone, and MIC_BA_ was the MIC of compound B in combination with compound A. FIC_A_ and FIC_B_ were the fractional inhibitory concentrations of compounds A and B, respectively. The interpretation of FICI values was as follows: FICI ≤ 0.5: Synergistic; 0.5 < FICI ≤ 1: Partial synergy; 1 < FICI ≤ 2: Indifferent; FICI > 2: Antagonistic.

### Bacterial growth curve assay

The bacterial growth curve assay was performed with minor modifications from previous studies (45). Bacterial cultures were diluted to an OD600 of 0.05, and 100 μL of bacterial suspension was added to a 96-well plate. 100 μL benzbromarone, colistin, and their combination were added to the wells. The plate was incubated at 37°C with shaking (200 rpm), and OD600 values were recorded every 2 h over an 18-h period to assess bacterial growth.

### Time kill curve determination assay

The growth inhibition effect of colistin in combination with benzbromarone was evaluated using time kill curve analysis (46). *K. pneumoniae* 12136, *A. baumannii* 50114, and *P. aeruginosa* 10147 were cultured overnight in MHB at 37°C with shaking (200 rpm). Cultures were diluted to approximately 1 × 10^6^ CFU/mL in fresh MHB.2.5 mL of bacterial suspension was added into 12 mL sterile shaking tube. Colistin (0.5 μg/mL), benzbromarone (64 μg/mL), or their combination (colistin 0.5 μg/mL + benzbromarone 64 μg/mL) was added to the corresponding tubes. The total system is 5 mL. Untreated bacteria served as the control group. Tubes were incubated at 37°C. At predetermined time points (0, 2, 4, 6, 8, 10, 12 h), samples were taken, serially diluted, and plated on Mueller–Hinton agar. CFU were counted after overnight incubation to construct time kill curves. Three biological replicates were set for each sample.

### Hemolysis assay

The hemolytic activity of colistin alone and in combination with benzbromarone was determined using Defibrinated sheep erythrocytes. Fresh red cells were washed three times with sterile PBS buffer, and resuspended to 2% (v/v). Red cells suspensions were incubated with different concentrations of colistin (0–64 μg/mL) in the presence or absence of benzbromarone (64 μg/mL) at 37°C for 2 h. Positive and negative controls: 0.1% Triton X-100 was used as the positive control (100% hemolysis), and PBS as the negative control. Measurement: After incubation, samples were centrifuged (1,000×*g*, 5 min), and the supernatant absorbance was measured at 543 nm using a multifunctional microplate reader (SPARK 10 M, PE, USA). Hemolysis (%) was calculated as: Hemolysis rate (%) = (A_sample_ − A_PBS_)/(A_Triton_ − A_PBS_) × 100. Three biological replicates were set for each sample.

### Cytotoxicity assay

The cytotoxicity of colistin, alone or combined with benzbromarone, was assessed in HepG2 (ATCC HB-8065) and Vero (ATCC CCL-81) cells HepG2 and Vero cells were cultured in Dulbecco’s Modified Eagle Medium (DMEM, Gibco, USA) supplemented with 10% fetal bovine serum (FBS) and 1% penicillin–streptomycin at 37°C in a humidified 5% CO_2_ incubator. Cells were seeded into 96-well plates at a density of 1 × 10^4^ cells/well and incubated overnight to allow attachment. Cells were exposed to increasing concentrations of colistin (0–64 μg/mL), with or without benzbromarone (64 μg/mL), for 24 h. Cell viability was assessed using the CCK-8 assay (Beyotime, Shanghai). After treatment, 10 μL of CCK-8 reagent was added to each well and incubated for 2 h. Absorbance was measured at 450 nm using a multifunctional microplate reader (SPARK 10 M, PE, USA). Cell viability (%) was expressed relative to untreated controls.

### NPN uptake assay

The NPN uptake assay was performed with slight modifications based on previous studies (46). N-Phenyl-1-naphthylamine (NPN) was a hydrophobic fluorescent probe with weak fluorescence in aqueous solutions but exhibited a significant fluorescence increase upon entering the bacterial outer membrane (47). This assay was used to evaluate the effects of drugs on the outer membrane permeability of Gram-negative bacteria. Briefly, bacterial cultures were grown to the logarithmic phase (OD600 ≈ 0.5), washed twice with PBS buffer, and adjusted to OD600 = 0.5. Benzbromarone, colistin, and their combination were added, followed by incubation at 37°C for 1 h. NPN (10 μM) was then added, and samples were incubated at 37°C for 5 min. Fluorescence intensity was measured using a multifunctional microplate reader (SPARK 10 M, PE, USA) with an excitation wavelength of 350 nm and an emission wavelength of 420 nm.

### PI uptake assay

The propidium iodide (PI) uptake assay was performed with minor modifications based on previous studies (48, 49). PI was a fluorescent probe that cannot penetrate intact bacterial membranes but enters cells when the inner membrane was compromised, binding to DNA and emitting fluorescence. This assay was used to assess bacterial inner membrane damage. Bacterial cultures were adjusted to OD600 = 0.5 and washed twice with PBS buffer. Cells were incubated at 37°C with benzbromarone, colistin, or their combination for 1 h. PI was then added at a final concentration of 10 μg/mL and incubated in the dark for 15 min. Fluorescence intensity was measured using a multifunctional microplate reader (SPARK 10 M, PE, USA) with an excitation wavelength of 535 nm and an emission wavelength of 617 nm. Subsequently, SYTO9 fluorescent dye (5 μM) was added and incubated in the dark for 15 min. The bacterial sample was then dropped onto a glass slide and observed under a confocal laser scanning microscope (N-STORM, Nikon, Japan). Green fluorescence represented live bacteria, and red fluorescence represented dead bacteria.

### Intracellular ROS measurement

ROS measurement assay was performed with minor modifications based on previous study (46). Bacterial cultures were adjusted to OD600 = 0.5 and washed twice with PBS buffer. Cells were incubated at 37°C with benzbromarone, colistin, or their combination for 1 h. Cells were exposed to the indicated drugs for 30 min at 37°C. 2′,7′-dichlorodihydrofluorescein diacetate (DCFH-DA, 10 μM) (Beyotime, Shanghai) was added and incubated 30 min in the dark. After PBS wash, fluorescence was measured at Ex 488 nm/Em 525 nm. Three biological replicates were set for each sample.

### Bacterial ATP level detection

The assay was performed according to previous study. Samples were grouped and processed as described previously (46). After 1 h drug treatment at 37°C, cells were pelleted and lysed according to the ATP assay kit (bioluminescence) instructions (Beyotime, Shanghai). Equal volumes of cell lysate and ATP working reagent were mixed in black 96-well plates and luminescence was recorded after 5 min. Three biological replicates were set for each sample.

### Scanning electron microscopy

Drug-treated and control cells were fixed in 2.5% glutaraldehyde at 4°C overnight, washed with PBS, and post-fixed with 1% osmium tetroxide for 1 h. Samples were dehydrated through graded ethanol (30–100%), critical-point dried, mounted on aluminum stubs, and sputter-coated with gold. Images were acquired on a field-emission SEM (SU-8010, Hitachi, Japan) at 3.0 kV (scale bar 2 μm).

### Galleria mellonella infection model

A Galleria mellonella larval infection model was used to preliminarily evaluate the protective effects of benzbromarone in combination with colistin (50, 51). Dormant G. mellonella larvae were surface-sterilized by gently wiping them with 75% ethanol, placed in petri dishes lined with filter paper, and incubated at 30°C for 3 h to allow awakening. Treated larvae were randomly divided into five groups (*n* = 10 per group) and labeled accordingly. Bacterial cultures were grown to the logarithmic phase (OD600 = 0.6), washed twice with sterile PBS buffer. The bacterial suspension was homogenized by repeated pipetting with a sterile insulin syringe to ensure uniform distribution. The bacterial solution was adjusted to OD600 ≈ 1.0 in PBS buffer. Each larva was injected with 10 μL of bacterial suspension into the hemocoel via the last left proleg using a sterile insulin syringe. Four groups of larvae were infected with 10^6^ CFU per larva, while a blank control group received an equal volume of PBS buffer. After 2 h of infection, drug treatments were administered. Benzbromarone, colistin, and their combination were injected into the hemocoel via the last left proleg using a sterile insulin syringe (10 μL per larva). The benzbromarone monotherapy group received 5 mg/kg per larva, while the colistin monotherapy group received 5 mg/kg per larva. The combination therapy group received 5 mg/kg benzbromarone and 2.5 mg/kg colistin per larva. The blank control group and the untreated group were injected with the same volume of PBS buffer containing 1% DMSO. Larvae were incubated at 30°C for 5 days, and survival was monitored every 24 h. The results were recorded, and survival curves were plotted. Live larvae appeared milky white, whereas dead larvae exhibited darkened bodies.

### Statistical analysis

All experiments were performed at least in triplicate, and data were expressed as mean ± standard deviation (Mean ± SD). Statistical analysis was conducted using GraphPad Prism 8.0. Independent sample comparisons were analyzed using an unpaired two-tailed t-test or one-way ANOVA. Statistical significance was defined as follows: *p < 0.05: Significant difference; **p < 0.01: Highly significant difference; ***p < 0.001: Very highly significant difference; ns: No significant difference.

## Data Availability

The original contributions presented in the study are included in the article/Supplementary material, further inquiries can be directed to the corresponding author.
